# Associations between breast milk intake volume, macronutrient intake and infant growth in a longitudinal birth cohort: the Cambridge Baby Growth and Breastfeeding Study (CBGS-BF)

**DOI:** 10.1017/S0007114522003178

**Published:** 2023-07-14

**Authors:** Laurentya Olga, Jacques Vervoort, Janna A. van Diepen, Gabriele Gross, Clive J. Petry, Philippa M. Prentice, Maciej Chichlowski, Eric A. F. van Tol, Ieuan A. Hughes, David B. Dunger, Ken K. Ong

**Affiliations:** 1 Department of Paediatrics, University of Cambridge, Cambridge, UK; 2 Department of Agrotechnology and Food Sciences, Wageningen University, Wageningen, the Netherlands; 3 Medical and Scientific Affairs, Reckitt/Mead Johnson Nutrition Institute, Nijmegen, the Netherlands; Evansville, IN, USA; 4 Institute of Metabolic Science, University of Cambridge, Cambridge, UK; 5 MRC Epidemiology Unit, Wellcome Trust-MRC Institute of Metabolic Science, NIHR Cambridge Comprehensive Biomedical Research Centre, Cambridge Biomedical Campus, University of Cambridge, Cambridge, CB2 0QQ, UK

**Keywords:** Breast milk, Breast-feeding, Infant growth, Macronutrient, Intake, Nutrition, Adiposity, Weight gain, Early life

## Abstract

Growth patterns of breastfed infants show substantial inter-individual differences, partly influenced by breast milk (BM) nutritional composition. However, BM nutritional composition does not accurately indicate BM nutrient intakes. This study aimed to examine the associations between both BM intake volumes and macronutrient intakes with infant growth. Mother–infant dyads (*n* 94) were recruited into the Cambridge Baby Growth and Breastfeeding Study (CBGS-BF) from a single maternity hospital at birth; all infants received exclusive breast-feeding (EBF) for at least 6 weeks. Infant weight, length and skinfolds thicknesses (adiposity) were repeatedly measured from birth to 12 months. Post-feed BM samples were collected at 6 weeks to measure TAG (fat), lactose (carbohydrate) (both by ^1^H-NMR) and protein concentrations (Dumas method). BM intake volume was estimated from seventy infants between 4 and 6 weeks using dose-to-the-mother deuterium oxide (^2^H_2_O) turnover. In the full cohort and among sixty infants who received EBF for 3+ months, higher BM intake at 6 weeks was associated with initial faster growth between 0 and 6 weeks (*β* + se 3·58 + 0·47 for weight and 4·53 + 0·6 for adiposity gains, both *P* < 0·0001) but subsequent slower growth between 3 and 12 months (*β* + se − 2·27 + 0·7 for weight and −2·65 + 0·69 for adiposity gains, both *P* < 0·005). BM carbohydrate and protein intakes at 4–6 weeks were positively associated with early (0–6 weeks) but tended to be negatively related with later (3–12 months) adiposity gains, while BM fat intake showed no association, suggesting that carbohydrate and protein intakes may have more functional relevance to later infant growth and adiposity.

Early postnatal nutrition strongly affects or mediates the link between early-life and long-term health outcomes^(1)^. With regard to this, breast-feeding (BF) has been associated with desirable infancy growth patterns^(2)^ and reduced future metabolic risks^(3)^.

Compared with infants receiving formula, breastfed infants have been described to display remarkably different growth trajectories^(4)^, with faster weight and length gains in the first months^(4–6)^, an earlier infant BMI peak^(7)^, and slower growth gains up to 2–3 years^(4–6)^. On the contrary, infants receiving formula have slower beginning growth gains but the pace constantly increases, resulting in higher weight (both weight-for-age and weight-for-length *Z*-scores) compared with breastfed infants observed as early as 6 months of age^(6,8)^. Besides being heavier, infants receiving formula are characterised by larger skinfold thicknesses and higher body fat percentage from 9 months through 2 years of age^(8,9)^. Moreover, the growth rate moderating effect provided by BF (especially prolonged and exclusive BF) appears to persist^(8–10)^ and thus is associated with protection against childhood overweight/obesity^(3)^, particularly among those with higher risks, for example, born small/large for gestational age with rapid weight gain in early infancy^(11)^.

This overall growth trajectory of breastfed infants may be influenced by the composition of human breast milk (BM)^(12)^. Several studies have highlighted the potential importance of macronutrient composition in BM for weight and adiposity gains during infancy^(12–14)^. However, these strictly observational studies relied mainly on maternal recall and were confounded by many factors, including feeding behaviours, weaning/complementary feeding and maternal/pregnancy/infant co-morbidities^(15)^. Furthermore, the concentration of nutrients in BM does not reflect the absolute nutrient amounts consumed by infants. Quantification of the nutrients consumed from BM would therefore provide a better mechanistic link between BF and infancy growth and adiposity.

In this study, we aimed to measure macronutrient intake from BM by quantifying carbohydrate, fat and protein concentrations from BM combined with estimating the volume of BM consumed by infants in a strictly monitored longitudinal UK-based birth cohort. We hypothesised that the amount of BM intake and specific BM composition are associated with weight and adiposity gains during infancy.

## Methods

### Study design and participants

The Cambridge Baby Growth and Breastfeeding Study (CBGS-BF)^(16)^ comprises in total ninety-four mother–infant dyads who were consecutively recruited at birth from one maternity hospital in Cambridge, UK (2015–2017). These inclusion criteria were applied: singleton pregnancy, no significant maternal illness (including hypertension and/or diabetes during pregnancy and any other chronic illnesses, or any regular medication use), full-term vaginal delivery, maternal pre-pregnancy BMI within the healthy range^(17)^ and intention to exclusive breast-feeding (EBF) for at least 6 weeks. The protocol of the study has been published elsewhere^(16)^. Given the strict inclusion criteria of the study, the sample size was determined by the feasibility of recruitment with a minimal number of samples of 67, aiming to detect a minimal effect size of 0·2 with 90 % power at the 5 % level.

The total numbers of subjects with BM intake volume (main outcome thus being the analytic sample) and BM macronutrient concentrations measurements were 70 and 59, respectively. In total, there were forty-seven subjects with complete measurements (online Supplementary Table 1).

This study was conducted in compliance with the Declaration of Helsinki. All procedures involving research participants in the study were approved by the National Research Ethics Service Cambridgeshire 2 Research Ethics Committee (reference number 11/EE/0068. Written informed consent was obtained from all mothers in the study, for themselves and on behalf of their infants.

### Anthropometry

Trained paediatric research nurses measured infant weight, length and skinfold thickness at four sites (triceps, subscapular, flank and quadriceps). Following UK guidelines, weight, length and BMI values were converted to sex- and age-adjusted standard deviation scores (SDS) using the British 1990 growth reference at birth (LMS Growth^(18)^) and subsequently using WHO International Growth Standard (‘anthro’ package-WHO^(19)^). Internal SDS were calculated for each skinfold thickness site by employing the residuals from linear regression models, adjusted for sex and age, and then the mean skinfolds SDS was calculated as measures of infant adiposity.

Anthropometry data quality control was maintained by involving the same paediatric research nurses throughout the study, regular machine calibration, and periodical personnel training and measurement review/monitoring conducted by an anthropometry specialist in the unit.

### Breast milk intake volume measurement

The volume of BM consumed by each infant at 4–6 weeks of age was measured using the dose-to-the-mother deuterium-oxide (^2^H_2_O) turnover technique, and the detailed methodology has been described previously^(16,20)^.

In brief, when infants were 4 weeks old, baseline urine samples were taken from both mothers and infants. The next day, mothers ingested 50 g of deuterium-enriched (tracer) water, which was then incorporated into BM and passed to the infants during BF. Repeated urine samples were collected from both mothers and infants over the following 2 weeks. To collect urine samples from infants, cotton wool was placed into the nappies and checked every hour. Cotton wool that was saturated with urine was transferred to a syringe barrel, and urine was extracted using the syringe plunger.

^2^H is a non-radioactive, naturally occurring isotope. In this study, ^2^H enrichment in the urine samples was measured using isotope-ratio MS. The amount of BM intake volume was estimated by formulating a curve of isotope enrichment transfer between each mother and her infant^(16,20)^.

### Breast milk collection and macronutrient assays

Mothers were asked to hand or pump express their BM samples after feeding their infants at each visit from birth until 12 months of infant age (if mothers were still BF)^(16)^. Expression was done from the same breast last used to feed the infants. Individual samples were kept frozen at -70^°^C until processed for further analyses at a single time point. Of note, as reported in the literature, post-feed samples may contain higher fat^(21)^ and total protein^(22)^ and therefore may not be able to represent the exact estimates of macronutrient intakes in BM.

At the time of assay, individual BM samples were defrosted and thoroughly mixed. From these homogenised BM samples, lactose (as the predominant BM carbohydrate) and TAG (as the predominant BM fat) concentrations were measured by ^1^H-NMR^(12,14)^. Total nitrogen was measured by the Dumas method to calculate BM protein concentration^(23)^. These BM macronutrient assays were based on our previous work with CV of 0·3–5·8 % for lipids and 0·4–4·7 % for polar metabolites (e.g. lactose). These values have been reported in detail in our previous publications^(12)^.

Since BM intake volume is not significantly different between the age of 1 and 6 months^(24)^, we used the single BM intake volume measurement at the age of 6 weeks as an estimate for other time points, that is, 6 weeks, 3 months and 6 months to enable longitudinal BM macronutrient intake analyses.

### Calculations and statistical analyses

Atwater conversions were used to calculate the metabolisable energy concentration of BM, taking energy concentrations of 4, 9 and 4 kcal/g for lactose, fat and protein, respectively^(25)^. BM macronutrient intake (g/d) was calculated as the product of each macronutrient concentration (g/100 ml) at the age of 6 weeks – 6 months and BM intake volume (l/d) at 6 weeks as proxy for all other time points.

Continuous variables were summarised as mean and standard deviation or median (interquartile range), and categorical variables as number (%). The analytic sample (*n* 70) did not have any missing anthropometry or maternal/perinatal data. Subjects without BM macronutrient concentrations measured were not included in the relevant analyses (pairwise deletion method to handle missing data).

Multiple linear regression models were run with BM intake volume and BM macronutrient intakes as predictors and infant growth gains as outcomes. Prior to this, both BM macronutrient concentrations and intakes were found unrelated to maternal age, ethnicity, pre-pregnancy BMI, height and parity from correlation analyses; therefore, these factors were not included as covariates in the regression models. Taking these preliminary analyses into account and using a data-agnostic approach while also considering biological plausibility, those regression models were further adjusted for infant sex, birth weight SDS, GA, postnatal age at visit and EBF status. To demonstrate robust associations between BM macronutrient intakes and subsequent infant growth outcomes, these regression analyses were performed *only* among infants who received EBF for 3+ months (*n* 40, online Supplementary Table 1).

To capitalise on the longitudinal growth and macronutrient intakes data with appropriate handling of missing values, linear mixed-effects models were used to examine the associations between growth and body composition parameters, that is, weight, height, BMI and mean skinfolds, with each BM macronutrient intake, that is, carbohydrate, protein and fat. The models were adjusted for the same set of covariates as the multiple linear regressions. Due to non-linear relationships between growth and age (as reported previously^(26,27)^), time was modelled using linear splines with one knot at the age of 3 months, resulting in two periods: 0–3 and 3–12 months. Models were fitted to the data by restricted maximum likelihood.

All analyses were performed using SPSS version 25 (IBM Corp.) and R version 3.6.1 (R Foundation for Statistical Computing). *P* < 0·05 indicated statistical significance in descriptive statistics. Bonferroni corrections were used to take into account the multiple comparisons, assuming 2X broad growth phenotypes (adiposity and length) and 3X time periods for multiple linear regression models (i.e. *P* < 0·05/6 = 0·0083) or 2X time periods for linear mixed-effects models (i.e. *P* < 0·05/4 = 0·0125).

## Results

All seventy infants (analytic sample) were EBF for at least 6 weeks. After that, ten commenced mixed feeding with the introduction of formula between 6 and 12 weeks, while the remaining sixty (86 %) were EBF for 3+ months, of which thirty-eight were EBF for the first 3–6 months and twenty-two infants were EBF for 6+ months. Their characteristics are summarised in Table 1. All mothers had BMI values that were within the healthy range^(17)^ (mean + sd: 22·54 + 2·71). Almost 40 % were primiparous and most (94 %) were of White/European ethnicity. Their baseline demographics did not differ from the whole study population (*n* 94; online Supplementary Table 2).

**Table 1.** Baseline characteristics of the analytic study sample (*n* 70)

Characteristics	*n*	Mean	sd
Maternal			
Age at delivery (years)	70	33·57	4·3
Pre-pregnancy BMI (kg/m^2^)	70	22·54	2·71

		*n*	%
Parity (primiparous)	70	26	37·1
Ethnicity (White/European ethnicity)	70	66	94·3
Infants			
Birth			
Sex (male)	70	41	58·6

		Mean	sd
Gestational age (weeks)	70	40·36	1·08
Weight (kg)	70	3·63	0·43
Weight-SDS	70	0·14	0·76
Length (cm)	70	51·29	1·8
Length-SDS	70	−0·18	0·74
BMI (kg/m^2^)	70	13·78	1·16
BMI-SDS	70	0·09	0·92
6 weeks			
Weight (kg)	70	5·06	0·66
Weight-SDS	70	0·22	0·97
Length (cm)	70	56·35	2·14
Length-SDS	70	0·12	1·02
BMI (kg/m^2^)	70	15·89	1·37
BMI-SDS	70	0·21	0·96
3 months			
Weight (kg)	70	6·12	0·83
Weight-SDS	70	0·01	1·05
Length (cm)	70	60·6	2·21
Length-SDS	70	0·09	1·03
BMI (kg/m^2^)	70	16·61	1·52
BMI-SDS	70	−0·05	0·99
12 months			
Weight (kg)	70	9·67	1·17
Weight-SDS	70	0·19	0·96
Length (cm)	70	75·25	2·6
Length-SDS	70	−0·004	1·0
BMI (kg/m^2^)	70	17·04	1·41
BMI-SDS	70	0·27	0·94

SDS, standard deviation scores.

Values are mean and sd or *n* (%) as appropriate.

SDS values at birth are based on British 1990 growth reference and at other time points are based on WHO International Growth Standard.

### Breast milk intake volumes

The values of BM intake volume measured at 4–6 weeks ranged from 0·45 to 1·26 (mean + sd 0·78 + 0·16) l/d and were higher in male *v*. female infants (0·81 + 0·17 *v*. 0·73 + 0·14, respectively, *P* = 0·044). When corrected for body size, mean and standard deviation of BM intake volume in this study is 0·15 + 0·02l/kg body weight/d. Compared with infants who continued EBF for 6–12 weeks, EBF infants for 3+ months consumed higher volumes of BM daily (0·8 + 0·16 *v*. 0·66 + 0·13, *P* = 0·017).

**Fig. 1.**
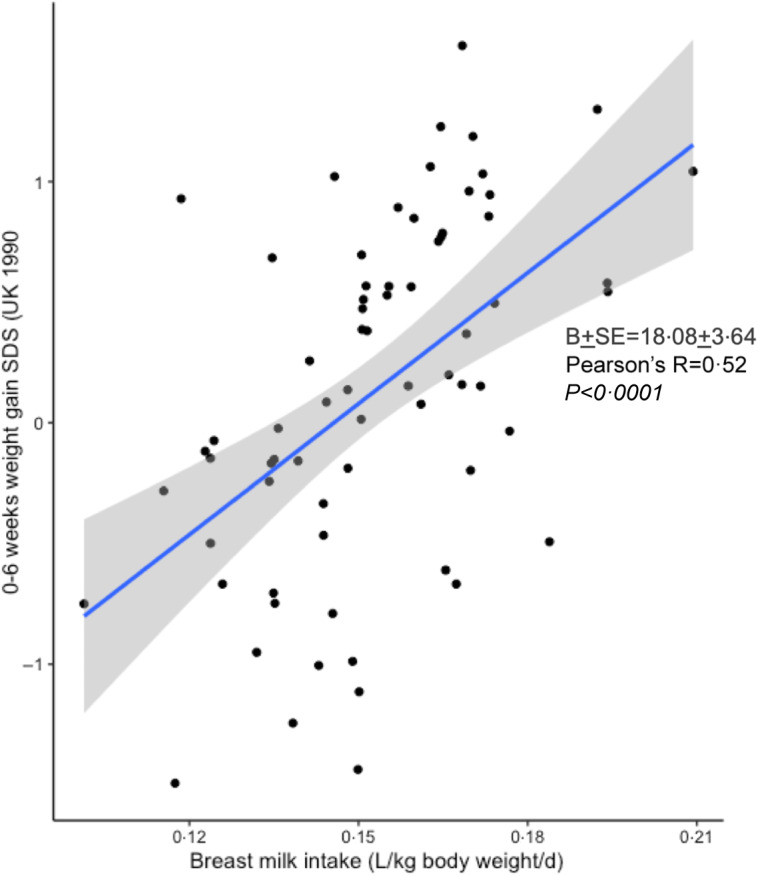
Correlation between BM intake volume at 4–6 weeks and infant weight gain from 0 to 6 weeks. Two-tailed partial correlation coefficient is presented, adjusted for infant sex and GA. BM, breast milk; GA, gestational age; SDS, standard deviation scores.

Infants with higher BM intake volumes at 4–6 weeks showed faster weight (adjusted Pearson’s R correlation coefficient = 0·71, *P* < 0·0001; Fig. 1), height and adiposity gains between birth and age 6 weeks (while *all* infants received EBF). Conversely, subsequent growth displayed the opposite correlations: infants with higher BM intake volumes at 4–6 weeks showed slower weight and adiposity gains between 3 and 12 months of age (online Supplementary Table 3). Similar associations were identified among the sixty infants who were EBF for 3+ months: higher breast milk intake volumes at 4–6 weeks predicted slower weight gain between 3 and 12 months (Table 2).

**Table 2.** Associations between BM macronutrient intake at 6 weeks with infant growth and adiposity

Outcomes (changes in SDS)				Predictors								
				BM macronutrient intake at 6 weeks								
	BM intake volume (l/d)			Carbohydrate (g/d)			Fat (g/d)			Protein (g/d)		
	*β*	se	*P*	*β*	se	*P*	*β*	se	*P*	*β*	se	*P*
Weight gain												
0–6 weeks	3·39	0·43	**<0·001** *	0·04	0·01	**<0·001** *	0·01	0·01	0·3	0·22	0·04	**<0·001** *
6 weeks–3 months	0·16	0·31	0·6	0·001	0·01	0·78	0·001	0·004	0·88	0·03	0·03	0·34
3–12 months	−2·21	0·64	**0·001** *	−0·03	0·01	**0·025**	−0·005	0·01	0·6	−0·13	0·06	0·05
Length gain												
0–6 weeks	1·42	0·63	**0·03**	0·01	0·01	0·52	−0·02	0·01	**0·02**	0·11	0·06	0·08
6 weeks–3 months	0·22	0·44	0·62	0·01	0·01	0·17	0·01	0·01	0·01	0·05	0·04	0·27
3–12 months	−0·92	0·65	0·16	−0·02	0·01	0·15	−0·005	0·01	0·51	−0·06	0·06	0·29
BMI gain												
0–6 weeks	3·68	0·78	**<0·001** *	0·05	0·01	**0·002** *	0·03	0·01	**0·02**	0·22	0·08	**0·01**
6 weeks–3 months	−0·12	0·48	0·81	−0·01	0·01	0·4	−0·01	0·01	0·38	−0·01	0·05	0·91
3–12 months	−2·33	0·75	**0·003** *	−0·03	0·01	0·08	−0·003	0·01	0·81	−0·13	0·08	0·1
Skinfold gain												
0–6 weeks	4·53	0·6	**<0·001** *	0·06	0·01	**<0·001** *	0·02	0·01	0·1	0·28	0·07	**<0·001** *
6 weeks–3 months	−0·92	0·48	0·06	−0·01	0·01	0·38	−0·001	0·01	0·84	−0·05	0·05	0·32
3–12 months	−2·65	0·69	**<0·001** *	−0·04	0·01	**0·009**	−0·01	0·01	0·6	−0·19	0·07	**0·006** *

BM, breast milk; EBF, exclusive breast-feeding; SDS, standard deviation scores; *β* + se, unstandardised regression coefficient + standard error.

* Statistical significance after Bonferroni correction for multiple comparisons (*P* < 0·0083).

The models include only infants with EBF 3+ months (*n* 40 for macronutrients models and 60 for BM intake volume models). All multiple linear regression models were adjusted for infant sex, birth weight SDS, gestational age, postnatal age at visit, EBF status at 6 months and other BM macronutrient concentrations at 6 weeks (for macronutrients models only). Associations at *P* < 0·05 are indicated in bold.

### BM macronutrient concentrations and intakes

Table 3 shows BM macronutrient concentration at 6 weeks (expressed as calories/100 ml and percentage of total calorie concentration (%TCC)). Compared with data from an earlier CBGS cohort^(12)^, the current BM samples contained significantly lower lactose, higher TAG and similar protein concentrations.

**Table 3.** BM macronutrient concentrations and intakes at 6 weeks

Macronutrients	BM macronutrient concentrations			
	Current study		Previous CBGS^(12)^ *	
	6 weeks		4–8 weeks	
	*n* 59		*n* 614	
	Mean	sd	Mean	sd
Carbohydrate (kcal/100 ml)	25·1	1·5	34·3	1·8
Carbohydrate (% TCC)	42·3	12·8	55·2	11·3
Fat (kcal/100 ml)	35·3	19·0	23·1	12·6
Fat (% TCC)	50·4	14·2	37·3	15·2
Protein (kcal/100 ml)	4·6	0·9	4·6	0·7
Protein (% TCC)	7·4	1·7	7·5	1·9
Total calorie concentration (kcal/100 ml)	64·98	19·48	61·8	13·04

Macronutrients	BM macronutrient intakes**			
	*n* 47			
	Mean	sd	Not available	
Carbohydrate (g/d)	50·8	11·7		
Fat (g/d)	29·6	17·4		
Protein (g/d)	9·1	2·1		
Total energy intake (kcal/d)	506·4	175·5		

BM, breast milk; CBGS-BF, the Cambridge Baby Growth and Breastfeeding Study; % TCC, % total calorie content (calculated as macronutrient energy/total energy concentration).

* The comparisons of carbohydrate and fat concentrations between current and previous studies reached significance (*P* < 0·05).

** BM intake volume was not available in the previous CBGS.

BM protein concentration was negatively associated with BM intake volume (Table 4). In addition, protein concentration was positively correlated with fat concentration, and both protein and fat were positively correlated with BM TCC.

**Table 4.** Correlations between BM intake volume (measured between 4–6 weeks) and macronutrient concentrations (measured at 6 weeks)

		Carbohydrate concentration (g/100 ml)	Fat concentration (g/100 ml)	Protein concentration (g/100 ml)	Total energy concentration (kcal/100 ml)
Intake volume (l/d)	*R*	0·14	−0·19	**−0·29**	−0·19
	*P*	0·3	0·2	**0·05**	0·2
Carbohydrate concentration (g/100 ml)	*R*	1	−0·14	−0·13	0·02
	*P*		0·4	0·4	0·9
Fat concentration (g/100 ml)	*R*		1	**0·66**	**0·997**
	*P*			**<0·001**	**<0·001**
Protein concentration (g/100 ml)	*R*			1	**0·69**
	*P*				**<0·001**

BM, breast milk.

Pearson’s *R* correlation coefficients and their corresponding *P* values are presented.

Statistically significant correlations (*P* < 0·05) are highlighted in bold.

### BM macronutrient intakes and infant growth over time

The associations between BM intake volume as well as macronutrient intakes and infant growth between 0 and 12 months were evaluated.

Separately at each time point, carbohydrate and protein intakes at 4–6 weeks were positively correlated with all early growth parameters, that is, at 6 weeks and 3 months (online Supplementary Table 4). In linear regression models, carbohydrate and protein intakes were positively associated with early gains in weight, BMI and adiposity between 0 and 6 weeks but tended to show inversely associations with later gains in weight, BMI and adiposity from 3 to 12 months, although only protein and change in skinfolds reached Bonferroni significance (Table 2). Linear mixed-effects models confirmed the positive associations between carbohydrate and protein intakes with early gains in weight between 0 and 3 months and showed a negative association between carbohydrate intakes with later weight between 3 and 12 months (Table 5).

**Table 5.** Longitudinal associations between BM macronutrient intake and infant growth and adiposity

Outcomes (changes in SDS)	Predictors								
	Carbohydrate (g/d)			Fat (g/d)			Protein (g/d)		
	Estimate	se	*P*	Estimate	se	*P*	*β*	se	*P*
Early infancy period: 0–3 months									
Weight SDS	0·02	0·01	**0·002** *	0·005	0·003	0·07	0·05	0·02	**0·01**
Length SDS	0·004	0·01	0·48	0·002	0·003	0·38	0·01	0·02	0·55
BMI SDS	0·02	0·01	**0·008** *	0·004	0·004	0·23	0·06	0·03	**0·04**
Mean SF SDS	0·01	0·01	0·2	0·001	0·004	0·85	0·02	0·03	0·41
Late infancy period: 3–12 months									
Weight SDS	−0·02	0·01	**0·03**	−0·003	0·004	0·44	−0·06	0·05	0·6
Length SDS	−0·02	0·01	**0·04**	−0·005	0·004	0·17	−0·03	0·04	0·45
BMI SDS	−0·02	0·01	0·15	−0·001	0·01	0·88	−0·02	0·05	0·74
Mean SF SDS	−0·03	0·01	0·08	−0·002	0·01	0·73	−0·05	0·06	0·38

BM, breast milk; SDS, standard deviation scores, SF, skinfolds; GA, gestational age; EBF, exclusive breast-feeding.

* Statistical significance after Bonferroni correction for multiple comparisons (*P* < 0·0125).

Fixed effect estimates +se are displayed.

Analyses are based on linear mixed-effect models, adjusted for infant sex, birth weight SDS, GA, postnatal age at visit, EBF status at 3 months and other BM macronutrient concentrations. Smoothing splines were added to the models with knot at 3 months. Associations at *P* < 0·05 are indicated in bold.

## Discussion

The objective of this study was to investigate the associations between BM macronutrient intakes and infant growth/adiposity and therefore require a well-controlled mother–infant dyad study population. In this cohort, all infants were vaginally born of healthy mothers with BMI in the healthy range and were EBF for the first 6 weeks. The prevalence of EBF at 3 months in this cohort (86 %) was much higher than the UK national prevalence in 2010 (17 %)^(28)^.

Infant sex and EBF duration influenced BM intake volumes, with males and longer EBF duration associated with higher BM intake volumes at 6 weeks of age. The effect of infant sex on BM intake volume reported in this study (male infants consumed 80 g more BM daily) was similar to a multinational study conducted by Da Costa *et al*. with 50 g/d more BM consumed by males than females^(29)^.

Compared with previous data from an earlier CBGS cohort^(12)^ and a published overview by Ballard and Morrow^(14)^, BM in this study contained lower lactose, higher fat, but comparable protein concentrations (term BM macronutrient concentrations according to the overview were^(14)^: 0·9–1·2 g/100 ml protein, 3·2–3·6 g/100 ml fat and 6·7–7·8 g/100 ml lactose). BM TCC was similar to previous reports: in this study BM at 6 weeks contained 65·0 + 19·5 kcal TCC/100 ml compared with values in the published overview (65–70 kcal/100 ml BM^(14)^). Lower lactose and higher fat BM contents observed in this study might be due to differences in BM sampling at distinct time points as compared with the pooled sampling over a 2-week period in the previous CBGS study^(12)^.

**Fig. 2.**
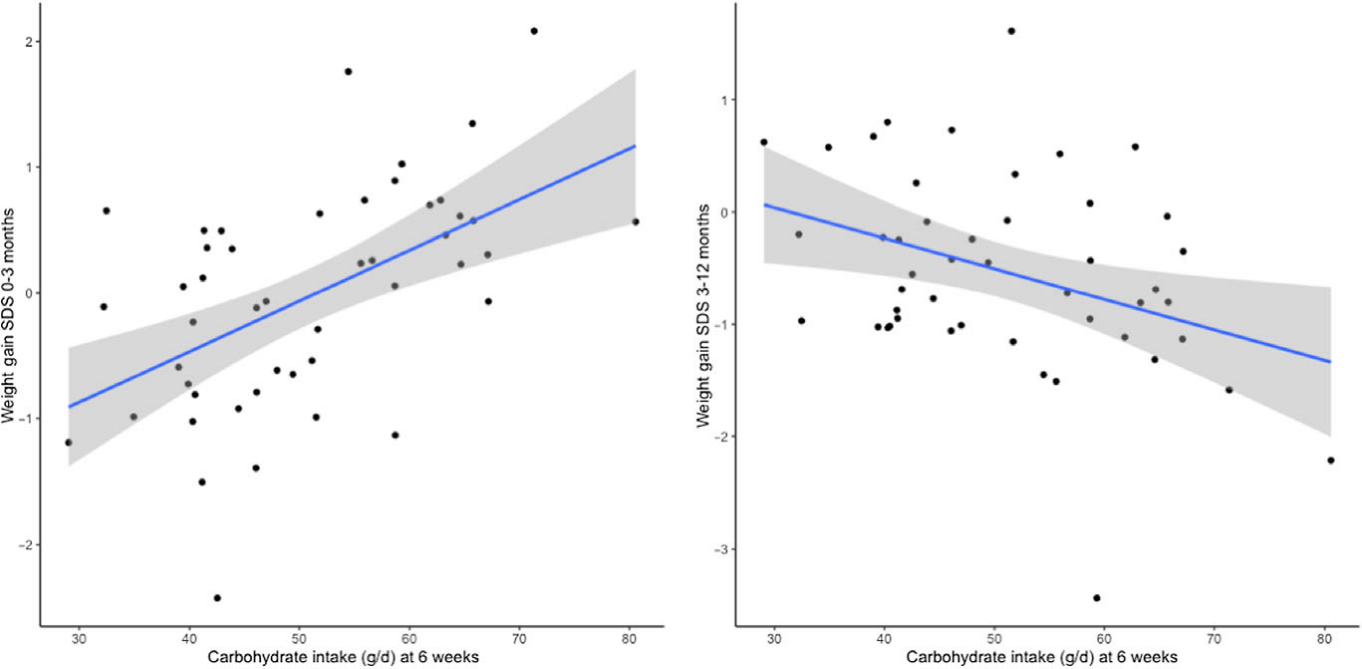
Correlation between carbohydrate intake at 6 weeks and infant weight gain. SDS, standard deviation scores.

It might be expected that higher BM intake volumes in EBF infants would be positively related to growth rates. Indeed, we observed that shortly after birth, infants with higher BM intake volumes at 4–6 weeks displayed faster gains in all growth parameters. However, at later time points, weight and adiposity gains were slower in these infants (Fig. 2, online Supplementary Table 2, Supplementary Fig. 1). This growth pattern is in accordance with the characteristics of breastfed infants: rapid growth gains during early infancy but slower in later months^(8)^. This slower growth pattern in later infancy is speculated to be protective against obesity risk in adolescence and adulthood^(30)^. The main objective of this study was to investigate which macronutrients of BM were related to this typical growth pattern of breastfed infants.

Of note, of all covariates included in the models, birth weight and gestational age were the most significant contributors to early weight gain 0–6 weeks (% variance explained 9 % and 3 %, respectively). As shown in Supplementary Fig. 1, the highest tertile BM intake group were born heavier compared with the other groups.

In our previous CBGS cohort, BM fat and carbohydrate concentrations were associated with changes in infancy weight and adiposity up to age 12 months; BM protein concentration was positively correlated with 12-month BMI^(12)^. However, data on milk intakes were not available in that cohort. In contrast, the current study design, including BM intake volume measurement, allowed for exploration of the effects of macronutrient intakes from BM on infancy growth and adiposity. Positive associations were identified between BM carbohydrate and protein intakes with weight and BMI during early infancy period, but negative trends were seen with later outcomes. However, no associations were found between BM fat intake and infant growth.

The positive associations between BM carbohydrate and protein intakes with earlier infant growth gains found in this study are in line with the associations of the concentrations of those macronutrients with early growth that have been reported in the existing literature^(12,31)^. The excess of BM carbohydrate intake could presumably be stored as glycogen and fat and therefore result in greater growth and increased adiposity^(32)^. To the best of our knowledge, the evidence linking protein intakes from BM with infant growth is still scarce, unlike studies involving formula. High protein intakes from traditional formula has been linked with rapid weight gain in comparison with both breastfed^(13)^ and infants fed with low-protein formula^(33)^. In this study, the independent positive association between BM protein intake with early infant growth was also not confounded by BM intake volume, since BM protein concentrations were inversely correlated with BM intake volume (Table 4), which is also consistent with previous observations^(34,35)^. In the Davis Area Research on Lactation, Infant Nutrition and Growth (DARLING) study, milk protein concentration was negatively correlated with milk volume at 6 and 9 months, while milk lactose was positively correlated^(34)^. Similar to our findings, the milk energy density in that study was highly correlated with lipid concentration. Furthermore, the robust positive association between protein intakes and early postnatal growth parameters that persisted after Bonferroni correction for multiple comparisons are consistent with observations linking high protein intake from formula with rapid weight gain^(13)^, possibly by promoting higher levels of insulin-like growth factor 1. Animal and human studies have reported that high protein intakes increase insulin-like growth factor 1 secretion and therefore may accelerate gains in both muscle and fat^(36,37)^.

Interestingly, inverse associations or trends were also observed between BM carbohydrate intake with later weight and adiposity gains, as well as between BM protein intake with later adiposity gain (Table 2). This might suggest that both macronutrients substantially contribute to the typical growth pattern of breastfed infants.

High intake of protein, especially if sustained in long duration, appears to help reduce food intake, body weight and body adiposity in many well-documented studies^(38–40)^. Therefore, increasing protein intake is also one of the established strategies to lose weight, especially to maximise fat loss while avoiding the detrimental loss of fat-free or muscle mass^(39)^. This is because dietary proteins are anabolic dietary compounds as their breakdown predominate over their synthesis^(39)^. In addition, proteins are considered to have a greater satiating effect than carbohydrate or fat, because protein intake could induce the release of satiety hormones from gastrointestinal tract, for example, cholecystokinin, peptide YY and glucagon-like peptide 1^(41)^. All of these could probably explain the mechanism behind the strong inverse relationship between BM protein intake and later skinfold gain between 3 and 12 months in this study.

The lack of associations between BM fat intake with infant growth was unexpected and inconsistent with previous studies which have suggested that greater BM fat content might promote infant satiety and hence may be beneficial in preventing rapid gains in infant weight and adiposity^(42,43)^. Further exploration using qualitative analyses of BM fat contents beyond only TAG, for example, BM SCFA composition, may provide a better understanding.

The main strength of this study is the measurement of BM intake volume that enables BM nutrient intakes calculation. There are previous studies investigating BM macronutrient intakes, but they usually estimated BM intake volume via 24-h test weighing (i.e. weighing infants before and after every feed for a 24-h period)^(44,45)^ during EBF period or using the values reported from the literature^(46)^. In this study, ^2^H-labelled water was perceived to provide more precise BM intake volume estimates compared with the other methods. Apart from that, this method is also non-invasive, does not require researcher’s intensive observation during the procedure, should not affect milk production or feeding pattern and is reported to highly correlate with 24-h test weighing^(47)^. As demonstrated here, BM intake volumes and BM nutrient intakes, rather than simply BM concentrations, are important factors contributing to the effects of BF on infant growth and therefore should be considered in future studies. In addition, longitudinal study design and analyses of specific growth periods are crucial to understanding nutrition-related factors affecting infant growth and weight/adiposity gains.

However, we recognise several limitations, especially the use of single milk sample per visit rather than a pooled sampling that can take into account sample variability as demonstrated in our previous study^(12)^. We assume the higher fat and lower lactose concentrations observed in this study were due to distinct time points during sampling. Second, only 31 % of the analytic sample (22 out of 70) continued EBF for at least 6 full months, while the remaining infants experienced substantially reduced exposure of BM before 6 months. This could bias towards the null associations between BM macronutrient intakes and growth that were analysed longitudinally until 12 months of age in this study. Moreover, although serial BM macronutrients were conducted, BM intake volume was only measured at a single point, that is, 6 weeks. Since most of our research participants were of White/European ethnicity, the applicability of the results of this study needs validation in larger and more diverse populations. The small number of subjects, especially those with complete measurements, reduced the power in statistical analyses, and therefore the results require confirmation. Comprehensive dietary evaluations of both mothers during EBF and infants during complementary feeding should also be included in future studies.

In conclusion, BF may affect immediate and subsequent infancy growth, and this effect could be modified by BM intake volumes. BM macronutrient intake, especially lactose and protein, may have functional relevance to infant growth and adiposity.

## Supplementary material

For supplementary material accompanying this paper visit https://doi.org/10.1017/S0007114522003178. click here to view supplementary material
