# Supplementary material for: Associations between breast milk intake volume, macronutrient intake and infant growth in a longitudinal birth cohort: the Cambridge Baby Growth and Breastfeeding Study (CBGS-BF)
Source: Br J Nutr. 2022 Oct 19;130(1):56–64. doi: 10.1017/S0007114522003178 (PMC10244014; doi:10.1017/S0007114522003178)
Supplement: Supplementary file 1 [file S0007114522003178sup001.docx]

**Supplementary materials**

Supplementary Table 1 **Number of cohort participants with measurements**

| **Parameters/measurements** | **Number of subjects** |
| --- | --- |
| Total recruited | 94 |
| Complete anthropometry measurements from 0-12 months | 88 |
| BM intake volume (analytic sample) | 70 |
| BM macronutrient concentrations | 59 |
| All measurements | 47 |
| With all measurements and received EBF for 3+ months | 40 |

Supplementary Table 2 **Comparison of baseline characteristics between analytic sample and the whole study population**

| **Characteristics** | **All subjects** | | **Analytic sample**  **(Subjects with BM intake volume)** | |
| --- | --- | --- | --- | --- |
|  | **N** | **Values** | **N** | **Values** |
| **Maternal** |  |  |  |  |
| Age at delivery, years | 94 | 33.25+4.68 | 70 | 33.57+4.3 |
| Pre-pregnancy BMI, kg/m^2^ | 92 | 22.27+2.55 | 70 | 22.54+2.71* |
| Height, cm | 91 | 166.74+6.21 | 69 | 166.8+6.44 |
| Parity (% primiparous) | 94 | 37(39.4%) | 70 | 26(37.1%) |
| Ethnicity (% White/European) | 94 | 86(91.5%) | 70 | 66(94.3%) |
| **Birth** |  |  |  |  |
| Sex (% male) | 94 | 57(60.6%) | 70 | 41(58.6%) |
| Gestational age, weeks | 94 | 40.26+1.06 | 70 | 40.36+1.08 |
| Weight (kg) | 94 | 3.61+0.42 | 70 | 3.63+0.43 |
| Weight-SDS | 94 | 0.12+0.75 | 70 | 0.14+0.76 |
| Length (cm) | 94 | 51.19+1.79 | 70 | 51.29+1.8 |
| Length-SDS | 94 | -0.2+0.74 | 70 | -0.18+0.74 |
| BMI (kg/m^2^) | 94 | 13.74+1.1 | 70 | 13.78+1.16 |
| BMI-SDS | 94 | 0.08+0.87 | 70 | 0.09+0.92 |
| **6 weeks** |  |  |  |  |
| Weight (kg) | 94 | 5.05+0.61 | 70 | 5.06+0.66 |
| Weight-SDS | 94 | 0.19+0.91 | 70 | 0.22+0.97 |
| Length (cm) | 94 | 56.37+2.04 | 70 | 56.35+2.14 |
| Length-SDS | 94 | 0.12+0.98 | 70 | 0.12+1.02 |
| BMI (kg/m^2^) | 94 | 15.84+1.27 | 70 | 15.89+1.37 |
| BMI-SDS | 94 | 0.17+0.89 | 70 | 0.21+0.96 |
| **3 months** |  |  |  |  |
| Weight (kg) | 93 | 6.12+0.77 | 70 | 6.12+0.83 |
| Weight-SDS | 93 | -0.005+0.99 | 70 | 0.01+1.05 |
| Length (cm) | 93 | 60.58+2.02 | 70 | 60.6+2.21 |
| Length-SDS | 93 | 0.04+0.97 | 70 | 0.09+1.03 |
| BMI (kg/m^2^) | 93 | 16.64+1.43 | 70 | 16.61+1.52 |
| BMI-SDS | 93 | -0.04+0.94 | 70 | -0.05+0.99 |
| **12 months** |  |  |  |  |
| Weight (kg) | 88 | 9.66+1.09 | 70 | 9.67+1.17 |
| Weight-SDS | 88 | 0.16+0.92 | 70 | 0.19+0.96 |
| Length (cm) | 88 | 75.09+2.42 | 70 | 75.25+2.6 |
| Length-SDS | 88 | -0.1+0.99 | 70 | -0.004+1.0 |
| BMI (kg/m^2^) | 88 | 17.09+1.38 | 70 | 17.04+1.41 |
| BMI-SDS | 88 | 0.3+0.92 | 70 | 0.27+0.94 |

Values are mean+SD or n(%) as appropriate

SDS values at birth are based on British 1990 Growth Reference and at other time points are based on WHO International Growth Standard

******p<0.05* against excluded subjects, i.e. subjects without BM intake volume

SDS=standard deviation scores

Supplementary table 3 **Correlations between BM intake volume at 4-6 weeks and infant growth rates**

|  | BM intake volume (L/day) | |
| --- | --- | --- |
|  | Adjusted Pearson R | *p* |
| Weight gain (delta SDS) | | |
| 0-6wk | 0.71 | ***<0.0001*** |
| 6wk-3mo | 0.1 | *0.444* |
| 3-12mo | -0.5 | ***<0.0001*** |
| Height gain SDS |  |  |
| 0-6wk | 0.32 | ***0.007*** |
| 6wk-3mo | 0.11 | *0.43* |
| 3-12mo | -0.33 | ***0.014*** |
| BMI gain SDS | | |
| 0-6wk | 0.51 | ***<0.0001*** |
| 6wk-3mo | -0.01 | *0.924* |
| 3-12mo | -0.44 | ***0.001*** |
| Mean skinfolds gain SDS | | |
| 0-6wk | 0.6 | ***<0.0001*** |
| 6wk-3mo | -0.23 | *0.082* |
| 3-12mo | -0.54 | ***<0.0001*** |

Two-tailed partial correlation coefficients are presented, adjusted for infant sex and gestational age

Statistically significant correlations (*p<0.05*) are highlighted in bold

*Supplementary Table 4* **Longitudinal associations between BM macronutrient concentration and infant growth and adiposity**

Fixed effect estimates +SE are displayed.

Outcomes are changes in growth SDS parameters over time. Weight, length, and BMI SDS values are according to UK 1990 growth reference, adjusted for sex, GA, and postnatal age at visit. SF SDS are internally derived from the larger cohort of Cambridge Baby Growth Study, adjusted for sex, GA, and postnatal age at visit. Mean SF SDS are calculated as the average of individual SF taken from 4 sites: triceps, subscapular, flank, quadriceps.

Predictors are each BM macronutrient concentration over time in g/100 mL.

Analyses are based on linear mixed-effect models, adjusted for infant sex, birth weight SDS, GA, postnatal age at visit, EBF status at 3 months, and other BM macronutrient concentrations. Smoothing splines were added to the models with knot at 3 months.

EBF=exclusive breastfeeding, GA=gestational age, SDS=standard deviation scores, SF=skinfolds

| Outcomes | Predictors | | | | | | | | |
| --- | --- | --- | --- | --- | --- | --- | --- | --- | --- |
|  | Carbohydrate (g/100mL) | | | Protein (g/100mL) | | | Fat (g/100mL) | | |
|  | Estimate | SE | *p* | Estimate | SE | *p* | B | SE | *p* |
| Early infancy period: 0-3 months | | | | | | | | | |
| Weight SDS | -0.05 | 0.1 | *0.7* | 0.1 | 0.08 | *0.24* | 0.02 | 0.01 | *0.23* |
| Length SDS | -0.08 | 0.12 | *0.49* | 0.15 | 0.08 | *0.07* | 0.02 | 0.01 | *0.09* |
| BMI SDS | 0.02 | 0.16 | *0.89* | 0.07 | 0.1 | *0.5* | 0.01 | 0.02 | *0.54* |
| Mean SF SDS | 0.12 | 0.15 | *0.44* | 0.02 | 0.1 | *0.81* | 0.004 | 0.01 | *0.8* |
| Late infancy period: 3-12 months | | | | | | | | | |
| Weight SDS | -0.03 | 0.3 | *0.94* | 0.15 | 0.24 | *0.53* | -0.01 | 0.03 | *0.69* |
| Length SDS | -0.35 | 0.32 | *0.27* | -0.03 | 0.02 | *0.9* | -0.05 | 0.03 | *0.08* |
| BMI SDS | 0.29 | 0.41 | *0.48* | 0.14 | 0.29 | *0.62* | 0.01 | 0.03 | *0.72* |
| Mean SF SDS | -0.09 | 0.39 | *0.83* | 0.12 | 0.28 | *0.66* | 0.01 | 0.03 | *0.7* |

Supplementary Table 5 **Associations between BM intake volume (measured between 4-6 weeks) and macronutrient concentrations (measured at 6 weeks)**

B+SE=unstandardized regression coefficient+standard error

Associations at *p<0.05* are indicated in bold

| Predictor: BM Macronutrient concentrations (g/100 mL) at 6 weeks | Outcome: BM intake volume (L/day) between 4-6 weeks | | |
| --- | --- | --- | --- |
|  | B | SE | *p* |
| Carbohydrate | 0.07 | 0.07 | *0.34* |
| Fat | -0.02 | 0.01 | *0.2* |
| Protein | -0.24 | 0.12 | ***0.05*** |

Supplementary table 6 **Associations between BM macronutrient intake and infant growth and adiposity (cross-sectional analyses)**

Only involving infants with exclusive BF period 3+ months (N=40 for macronutrients models and 60 for BM intake volume models)

All multiple linear regression models were adjusted for infant sex, birth weight SDS, gestational age, postnatal age at visit, EBF status at 6 months, and other BM macronutrient concentration at 6 weeks (for macronutrients models only)

B=unstandardized regression coefficient

Associations at *p<0.05* are indicated in bold

EBF=exclusive breastfeeding, SDS=standard deviation scores

| **Outcomes** | **Predictors** | | | | | | | |
| --- | --- | --- | --- | --- | --- | --- | --- | --- |
|  | **BM macronutrient intake** | | | | | | **BM intake volume (L/day)** | |
|  | **Carbohydrate (g/day)** | | **Fat (g/day)** | | **Protein (g/day)** | |  |  |
|  | **B** | ***p*** | **B** | ***p*** | **B** | ***p*** | **B** | ***p*** |
| **Weight-SDS** | | | | | | | | |
| 6 wk | 0.04 | ***<0.0001*** | 0.003 | *0.632* | 0.19 | ***<0.0001*** | 3.53 | ***<0.0001*** |
| 3 mo | 0.04 | ***0.001*** | 0.011 | *0.172* | 0.25 | ***<0.0001*** | 3.75 | ***<0.0001*** |
| 6 mo | 0.02 | *0.217* | 0.01 | *0.118* | 0.2 | ***0.006*** | 2.47 | ***0.004*** |
| 12 mo | 0.02 | *0.332* | 0.01 | *0.367* | 0.14 | *0.067* | 1.4 | *0.104* |
| **Length-SDS** | | | | | | | | |
| 6 wk | 0.02 | *0.088* | -0.003 | *0.651* | 0.1 | *0.11* | 2.1 | ***0.001*** |
| 3 mo | 0.03 | ***0.014*** | 0.01 | *0.358* | 0.16 | ***0.004*** | 2.19 | ***<0.0001*** |
| 6 mo | 0.01 | *0.648* | 0.004 | *0.561* | 0.13 | ***0.048*** | 1.58 | ***0.027*** |
| 12 mo | 0.01 | *0.243* | 0.002 | *0.785* | 0.11 | *0.075* | 1.26 | *0.103* |
| **BMI-SDS** | | | | | | | | |
| 6 wk | 0.04 | ***0.001*** | 0.01 | *0.45* | 0.17 | ***0.014*** | 3.25 | ***<0.0001*** |
| 3 mo | 0.04 | ***0.019*** | 0.01 | *0.271* | 0.2 | ***0.009*** | 3.43 | ***<0.0001*** |
| 6 mo | 0.02 | *0.222* | 0.01 | *0.114* | 0.16 | ***0.04*** | 2.04 | ***0.042*** |
| 12 mo | 0.01 | *0.607* | 0.01 | *0.318* | 0.11 | *0.212* | 0.85 | *0.345* |
| **Skinfold thickness SDS** | | | | | | | | |
| 6 wk | 0.42 | ***<0.0001*** | 0.004 | *0.948* | 1.58 | ***0.002*** | 4.21 | ***<0.0001*** |
| 3 mo | 0.29 | ***0.008*** | 0.09 | *0.181* | 1.7 | ***0.002*** | 3.46 | ***<0.0001*** |
| 6 mo | 0.09 | *0.339* | 0.11 | ***0.03*** | 0.9 | ***0.048*** | 1.12 | *0.104* |
| 12 mo | 0.13 | *0.276* | 0.06 | *0.406* | 0.67 | *0.261* | 0.6 | *0.469* |

Supplementary Figure 1 **Correlation between BM intake volume at 4-6 weeks and infant weight gain from 0-6 weeks**

Two-tailed partial correlation coefficient is presented, adjusted for infant sex and GA

GA=gestational age, SDS=standard deviation scores


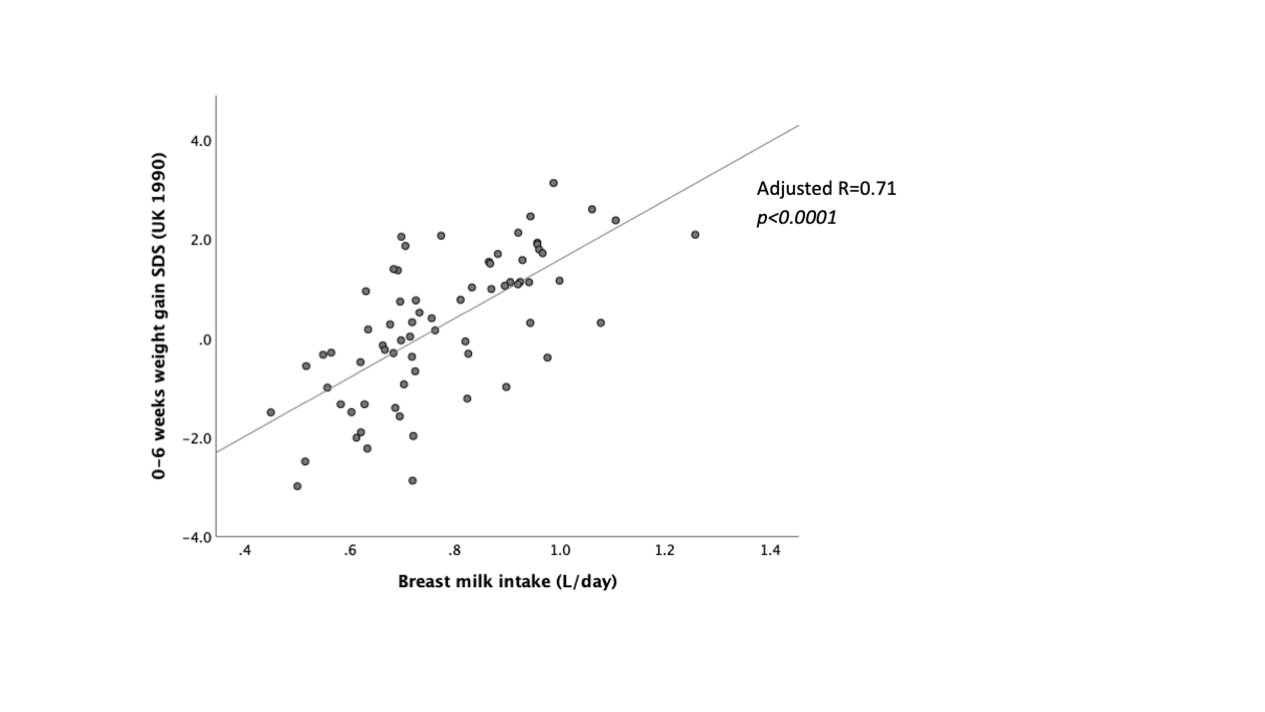


Supplementary Figure 2 **Weight trajectories between 0-12 months based on BM volume consumed by infants**

Only involving infants who were exclusively breastfed for at least 3 months (N=60)

BM intake volume tertiles (kg/day)
